# Cord Blood Methylmercury and Fetal Growth Outcomes in Baltimore Newborns: Potential Confounding and Effect Modification by Omega-3 Fatty Acids, Selenium, and Sex

**DOI:** 10.1289/ehp.1408596

**Published:** 2015-06-26

**Authors:** Ellen M. Wells, Julie B. Herbstman, Yu Hong Lin, Jeffery Jarrett, Carl P. Verdon, Cynthia Ward, Kathleen L. Caldwell, Joseph R. Hibbeln, Frank R. Witter, Rolf U. Halden, Lynn R. Goldman

**Affiliations:** 1 School of Health Sciences, Purdue University, West Lafayette, Indiana, USA; 2 Columbia Center for Children’s Environmental Health, Department of Environmental Health Sciences, Columbia University Mailman School of Public Health, New York, New York, USA; 3 Laboratory of Membrane Biochemistry and Biophysics, National Institute of Alcohol Abuse and Alcoholism, National Institutes of Health, Department of Health and Human Services, Rockville, Maryland, USA; 4 Division of Laboratory Sciences, National Center for Environmental Health, Centers for Disease Control and Prevention, Atlanta, Georgia, USA; 5 Department of Gynecology and Obstetrics, Johns Hopkins University School of Medicine, Baltimore, Maryland, USA; 6 Center for Environmental Security, Biodesign Institute, Global Security Initiative, Arizona State University, Tempe, Arizona, USA; 7 Department of Environmental and Occupational Health, Milken Institute School of Public Health, George Washington University, Washington, DC, USA

## Abstract

**Background:**

Methylmercury (MeHg) may affect fetal growth; however, prior research often lacked assessment of mercury speciation, confounders, and interactions.

**Objective:**

Our objective was to assess the relationship between MeHg and fetal growth as well as the potential for confounding or interaction of this relationship from speciated mercury, fatty acids, selenium, and sex.

**Methods:**

This cross-sectional study includes 271 singletons born in Baltimore, Maryland, 2004–2005. Umbilical cord blood was analyzed for speciated mercury, serum omega-3 highly unsaturated fatty acids (n-3 HUFAs), and selenium. Multivariable linear regression models controlled for gestational age, birth weight, maternal age, parity, prepregnancy body mass index, smoking, hypertension, diabetes, selenium, n-3 HUFAs, and inorganic mercury (IHg).

**Results:**

Geometric mean cord blood MeHg was 0.94 μg/L (95% CI: 0.84, 1.07). In adjusted models for ponderal index, βln(MeHg) = –0.045 (g/cm^3^) × 100 (95% CI: –0.084, –0.005). There was no evidence of a MeHg × sex interaction with ponderal index. Contrastingly, there was evidence of a MeHg × n-3 HUFAs interaction with birth length [among low n-3 HUFAs, βln(MeHg) = 0.40 cm, 95% CI: –0.02, 0.81; among high n-3 HUFAs, βln(MeHg) = –0.15, 95% CI: –0.54, 0.25; *p*-interaction = 0.048] and head circumference [among low n-3 HUFAs, βln(MeHg) = 0.01 cm, 95% CI: –0.27, 0.29; among high n-3 HUFAs, βln(MeHg) = –0.37, 95% CI: –0.63, –0.10; *p*-interaction = 0.042]. The association of MeHg with birth weight and ponderal index was affected by n-3 HUFAs, selenium, and IHg. For birth weight, βln(MeHg) without these variables was –16.8 g (95% CI: –75.0, 41.3) versus –29.7 (95% CI: –93.9, 34.6) with all covariates. Corresponding values for ponderal index were –0.030 (g/cm^3^) × 100 (95% CI: –0.065, 0.005) and –0.045 (95% CI: –0.084, –0005).

**Conclusion:**

We observed an association of increased MeHg with decreased ponderal index. There is evidence for interaction between MeHg and n-3 HUFAs; infants with higher MeHg and n-3 HUFAs had lower birth length and head circumference. These results should be verified with additional studies.

**Citation:**

Wells EM, Herbstman JB, Lin YH, Jarrett J, Verdon CP, Ward C, Caldwell KL, Hibbeln JR, Witter FR, Halden RU, Goldman LR. 2016. Cord blood methylmercury and fetal growth outcomes in Baltimore newborns: potential confounding and effect modification by omega-3 fatty acids, selenium, and sex. Environ Health Perspect 124:373–379; http://dx.doi.org/10.1289/ehp.1408596

## Introduction

Impaired fetal growth has been associated with neonatal morbidity and mortality (Ashworth 1998; Cheung et al. 2002). Additionally, the impact of fetal growth restriction has been shown to extend to chronic diseases of adulthood (Barker et al. 2002). There are several contributors to preterm birth and restricted fetal growth (Lang et al. 1996), including environmental exposures (Triche and Hossain 2007).

Methylmercury (MeHg), best recognized as a developmental neurotoxicant, may also impair fetal growth. This was initially demonstrated in settings with high MeHg exposures (Amin-Zaki et al. 1974; Harada 1978). More recently, Ramirez et al. (2000) reported an inverse association between gestational age at birth and cord blood mercury, and Xue et al. (2007) reported that maternal hair mercury was positively associated with very preterm birth (< 35 weeks), but not with all preterm births combined (< 37 weeks) (Ramirez et al. 2000; Xue et al. 2007). Mercury concentrations in hair and cord blood have also been associated with reduced birth weight (Foldspang and Hansen 1990; Lee et al. 2010; Ramón et al. 2009; Sikorski et al. 1986). Consistent with this, two longitudinal studies have reported associations of cord blood mercury with reductions of attained weight in early childhood (Grandjean et al. 2003; Kim et al. 2011). A significant association of cord blood mercury with reduced birth length was reported among a Spanish cohort (Ramón et al. 2009) and an association of maternal hair mercury with decreased head circumference at 20–24 weeks gestation among French infants with non-overweight mothers was reported (*p* < 0.06) (Drouillet-Pinard et al. 2010).

In contrast, some researchers report no association of cord blood mercury and/or maternal blood mercury with gestational age (Foldspang and Hansen 1990; Grandjean et al. 2001; Lederman et al. 2008), birth weight (Ding et al. 2013; Grandjean et al. 2001; Lederman et al. 2008), length (Ding et al. 2013; Lederman et al. 2008), or head circumference (Ding et al. 2013; Gundacker et al. 2010; Lederman et al. 2008). Two studies using biomarkers for inorganic mercury (IHg) exposure did not observe any association with birth outcomes (Bashore et al. 2014; Daniels et al. 2007). Additionally, there was a significant, positive bivariate correlation of maternal hair mercury with birth length in an Austrian cohort (Gundacker et al. 2010) and a significant, positive adjusted association of cord blood mercury with head circumference in a Filipino cohort (Ramirez et al. 2000). A French cohort reported significant positive associations of maternal hair mercury with birth weight and length, as well as borderline significant positive associations with head circumference among infants with overweight mothers (Drouillet-Pinard et al. 2010).

Interpretation of this literature is challenging due to the potential influence of confounders and mercury speciation on mercury’s effect on fetal growth. Seafood consumption is the main source of exposure to MeHg; however, this is also a major source for n-3 highly unsaturated fatty acids (HUFAs) and selenium, nutrients that enhance fetal growth (Choi et al. 2008). Use of total mercury (including IHg and MeHg) as a proxy for MeHg may result in overestimation of MeHg, particularly in populations with low fish consumption (Stern and Smith 2003). Because the toxicokinetics and target organs of MeHg and IHg differ (Clarkson and Magos 2006), this overestimation may reduce the ability of studies to identify associations with MeHg. Meanwhile, IHg itself is toxic, and it is possible that IHg exposure may result in toxicity in addition to MeHg (Chan 2011). Mercury toxicity may also be modified by sex (Gao et al. 2007; Goodrich et al. 2013). A recent review noted all of these concerns and recommended additional, careful investigations of MeHg toxicity (Karagas et al. 2012).

The present study was designed to address these concerns. Our goal is to determine the cross-sectional association between cord blood MeHg with measures of fetal growth, controlling for potential effects of co-exposure to n-3 HUFAs, selenium, and IHg, while assessing potential effect modification by sex.

## Methods

This research includes 271 infants from the Baltimore THREE Study (Tracking Health Related to Environmental Exposures), a cross-sectional birth study in Baltimore, Maryland, described in more detail in a previous publication (Apelberg et al. 2007). This research was in compliance with international human subject protection guidelines, and was approved by the Johns Hopkins School of Medicine Institutional Review Board (IRB). The IRB approved a waiver from the Health Insurance Portability and Accountability Act, and confirmed that it was not necessary to obtain informed consent because the study was determined to be of minimal risk, it only required collection of specimens which would otherwise be discarded, and all study data were anonymized following data collection. Analytical services provided by the U.S. Centers for Disease Control and Prevention (CDC) were determined to not constitute active engagement in research.

*Study population.* This study potentially included all singleton births delivered at the Johns Hopkins Hospital in Baltimore, Maryland, between November 2004 and March 2005. There were 597 singleton births (609 deliveries) during the study period. Umbilical cord blood was collected for 341 births; 300 had sufficient quantity for initial laboratory analyses that included mercury, and this is the Baltimore THREE Study population. For this publication, 29 additional births were excluded due to missing data for birth length (*n* = 5), maternal smoking (*n* = 1), serum selenium (*n* = 10), and serum fatty acids (*n* = 12); additionally, one infant was excluded due to an unusually small birth length. Thus, the sample size for this study is 271.

*Laboratory analyses.* Umbilical cord blood samples were collected, processed, and stored using standardized techniques (Witter et al. 2001). Samples were transferred to the CDC for metal and cotinine analyses.

Umbilical cord whole blood MeHg and IHg were determined using high performance liquid chromatography linked with inductively coupled plasma mass spectrometry (HPLC-ICP-MS) (Verdon et al. 2008). The limits of detection (LOD) were 0.48 μg/L (MeHg) and 0.75 μg/L (IHg). There were *n* = 47, 17.3% (MeHg) and *n* = 208, 76.8% (IHg) samples < LOD. A common method for addressing values < LOD is replacing them with a given value, such as LOD/2; however, this can introduce bias, particularly when the percentage of values < LOD is > 10%. Therefore, we requested additional data that were below the LOD but above the instrumentation LOD—these values were measured but too small to be declared significantly different from a blank. Although these values are notably less precise than values > LOD, they are likely more accurate than other commonly used imputation methods (Succop et al. 2004). There were *n* = 25, 9.2% (MeHg) and *n* = 130, 48.0% (IHg) values observed, yet < LOD; these values were used as replacements. This left only *n* = 22, 8.12% (MeHg) and *n* = 78, 28.9% (IHg) for which there was no information. These were replaced by the lowest observed value divided by the square root of 2. Total mercury was assessed using ICP-MS with an LOD of 0.33 μg/L; seven values were < LOD.

Umbilical cord serum selenium was determined using inductively coupled plasma dynamic reaction cell mass spectrometry (ICP-DRC-MS) (Xiao 2006). The LOD for selenium was 5 μg/L; all values were > LOD. Umbilical cord serum cotinine was measured using liquid chromatography in conjunction with atmospheric pressure ionization mass spectrometry (LC-API-MS) (Bernert et al. 1997). The LOD for cotinine was 0.015 ng/mL; 75 samples were < LOD. Cotinine was combined with reported smoking for analysis.

Cord serum fatty acids, including total n-3 HUFAs, were determined at the National Institute of Alcohol Abuse and Alcoholism using automated high-throughput extraction based on transesterification followed by fast gas chromatography (GC) (Lin et al. 2012). Samples were analyzed for common individual n-3 HUFAs as well as total n-3 HUFAs. All samples analyzed were above the detection limit for all analytes. However, 12 samples had insufficient serum for fatty acid analysis.

*Medical record abstraction.* Maternal smoking during pregnancy was classified as umbilical cord serum cotinine ≥ 10 ng/mL (Hukkanen et al. 2005) or smoking reported in the medical record. Prepregnancy body mass index (BMI) was calculated from height and prepregnancy weight as kilograms per meter squared. Missing data for prepregnancy weight (*n* = 3) and height (*n* = 7) were imputed using multivariable imputation (Wells et al. 2011). Hypertension includes chronic (pregestational) hypertension, gestational hypertension or preeclampsia, or hypertensive medication use. Diabetes includes chronic (pregestational) diabetes, gestational diabetes, or diabetic medication use. Small for gestational age was defined as < 10th percentile of growth among all infants of that gestational age; large for gestational age was defined as > 90th percentile; otherwise, infants are considered appropriate for gestational age. Ponderal index was calculated as [(birth weight; grams)/(birth length; centimeters)^3^] × 100.

*Data analysis.* Statistical analysis was performed with Stata 13.1. A *p*-value ≤ 0.05 was considered statistically significant, whereas *p*-values ranging from 0.05 to 0.10 were considered borderline statistically significant. The relationship of birth outcome with increasing MeHg was determined with multivariable linear regression models. Model covariates were determined by descriptive statistics (*p*-value ≤ 0.10 in bivariate comparisons of covariates with MeHg or birth outcome) and prior literature (selenium, n-3 HUFAs) (Choi et al. 2008). We considered including mode of delivery as a covariate in head circumference models; however, it was not a significant contributor to these models. IHg was evaluated as a potential confounder. Various forms for covariates, including dichotomous, categorical, linear, quadratic, and cubic splines, were explored visually using lowess curves and statistically by comparing Akaike Information Criterion and Bayesian Information Criterion values from models differing only by the covariate format. MeHg and IHg were lognormally distributed and therefore described using geometric means and natural-log transformed for regressions. Gestational age, birth weight, birth length, head circumference, and ponderal index were all evaluated as birth outcomes in regression models. Regression models are presented as adjusted only for gestational age; adjusted for demographics and medical history including gestational age (continuous), maternal age (quadratic), primiparity (dichotomous), prepregnancy BMI (continuous), maternal race (white/black/Asian), maternal smoking during pregnancy (dichotomous), maternal pregestational and gestational hypertension (dichotomous), maternal pregestational and gestational diabetes (dichotomous); or a fully adjusted model which also included umbilical cord serum selenium (continuous), umbilical cord serum n-3 HUFAs (continuous), and umbilical cord blood IHg (natural log transformed). We assessed model fit, heteroskedasticity, and influence. Variance inflation factors (VIF) were used to test for multicollinearity.

In a nontransformed regression equation, the expected change in *y* given a 1-unit change in *x* equals β(*x* + 1) – β(*x*). With a 1% change in a natural-log transformed *x* variable, this can be written as β[ln(*x* + 1%)] – β[ln(*x*)]; given the properties of natural logs, this can be rewritten as β{ln[(*x* + 1%)/*x*]}, which is equivalent to βln(101/100). For a 100% increase (approximately a doubling), this would be βln(200/100), which is roughly β(0.693).

Potential effect modification by infant sex, selenium, or n-3 HUFAs was investigated using stratified models (i.e., subgroup of males and subgroup of females) as well as fully adjusted models computing an interaction term across the entire cohort. In these analyses these three variables were dichotomized: sex (male/female), selenium (below the median/above the median), and n-3 HUFAs (below the median/above the median). Post hoc sensitivity analyses included determination of the individual impact of selenium, n-3 HUFAs, and IHg; comparison of models using total mercury versus MeHg; and analyses with IHg and MeHg as categorical variables.

## Results

Population characteristics and cord blood analytes are presented in Table 1. Average maternal age was 25.7 years [95% confidence interval (CI): 25.0, 26.5]. Mother’s average prepregnancy BMI was 26.4 kg/m^2^ (95% CI: 25.6, 27.3). Average n-3 HUFAs were 52.6 μg/mL (95% CI: 50.7, 54.4), and average selenium was 70.0 μg/L (95% CI: 68.5, 71.4). Geometric mean umbilical cord blood IHg was 0.13 μg/L (95% CI: 0.10, 0.17). Geometric mean umbilical cord blood MeHg was 0.94 μg/L (95% CI: 0.84, 1.07) and ranged from nondetectable levels to 15.4 μg/L. Cord blood MeHg was positively correlated with IHg, total Hg, and n-3 HUFAs, with Spearman’s ρ of 0.51, 0.91, and 0.23, respectively (see Supplemental Material, Table S1). MeHg was not correlated with serum selenium (Spearman’s ρ = –0.02).

**Table 1:** Population characteristics.

Characteristic/category	*n* (%)^*a*^	Cord blood MeHg (μg/L)^*b*^
Entire population	271 (100.0)	0.94 (0.84, 1.07)
Infant sex
Female	121 (44.7)	0.90 (0.74, 1.08)
Male	150 (55.4)	0.98 (0.84, 1.16)
Maternal age (years)
< 20	55 (20.3)	0.70 (0.54, 0.92)*
20–29	135 (49.8)	0.96 (0.83, 1.11)*
> 29	81 (29.9)	1.12 (0.86, 1.47)*
Maternal race
White/Caucasian	59 (21.8)	0.61 (0.44, 0.85)*
African American	190 (70.1)	1.00 (0.89, 1.13)*
Asian	22 (8.1)	1.79 (1.03, 3.11)*
Maternal smoking during pregnancy
None/passive	225 (83.0)	0.93 (0.81, 1.06)
Active smoker	46 (17.0)	1.02 (0.78, 1.34)
Maternal parity
Primiparous	116 (42.8)	0.93 (0.77, 1.13)
Not primiparous	155 (57.2)	0.95 (0.81, 1.12)
Maternal prepregnancy BMI (kg/m^2^)
< 25	142 (52.4)	0.98 (0.82, 1.17)
25–29.9	64 (23.6)	0.85 (0.66, 1.10)
≥ 30	65 (24.0)	0.96 (0.76, 1.21)
Hypertension^*c*^
No	244 (90.0)	0.97 (0.85, 1.10)
Yes	27 (10.0)	0.76 (0.48, 1.20)
Diabetes^*c*^
No	252 (93.0)	0.95 (0.83, 1.07)
Yes	19 (7.0)	0.91 (0.53, 1.58)
Cord serum n-3 HUFAs (quartiles) (μg/mL)
22.70–42.00	68 (25.1)	0.72 (0.56, 0.92)*
42.01–50.40	68 (25.1)	0.89 (0.71, 1.10)*
50.41–60.25	68 (25.1)	0.96 (0.75, 1.24)*
60.26–126.95	67 (24.7)	1.30 (1.01, 1.67)*
Cord serum selenium (quartiles) (μg/L)
42–62	73 (26.9)	0.90 (0.71, 1.15)
63–69	67 (24.7)	1.12 (0.89, 1.41)
70–78	67 (24.7)	0.85 (0.66, 1.09)
79–114	74 (23.6)	0.93 (0.72, 1.20)
Cord blood IHg (tertiles) (μg/L)
ND–0.08	91 (33.6)	0.54 (0.44, 0.65)*
0.09–0.57	92 (34.0)	0.91 (0.75, 1.10)*
0.58–2.17	88 (32.5)	1.76 (1.48, 2.10)*
ND, not detected. ^***a***^Percentages may not sum to 100 due to rounding. ^***b***^Values are geometric mean (95% CI). ^***c***^Includes pregestational and gestational. *p ≤ 0.05, one-way analysis of variance.		

Cord blood MeHg increased with increasing maternal age (Table 1). This association was borderline significant (defined as *p* ≤ 0.1 but > 0.05) based on an unadjusted linear regression model of maternal age (in years) and ln-transformed cord blood MeHg [βln(MeHg) = 0.019; 95% CI: –0.0002, 0.037]. Unadjusted regression model results indicated that infants born to black [βln(MeHg) = 0.50; 95% CI: 0.21, 0.78] or Asian [βln(MeHg) = 1.08; 95% CI: 0.59, 1.56] mothers had significantly higher MeHg levels than did children of white mothers. Higher MeHg was significantly related to higher n-3 HUFAs [βln(MeHg) = 0.02; 95% CI: 0.01, 0.02] and IHg (β = 0.22; 95% CI: 0.17, 0.28), but not selenium (β = –0.002; 95% CI: –0.01, 0.01), in unadjusted regression models.

Average measures of fetal growth and development are summarized in Table 2. There were *n* = 34 (12.5%) preterm (< 37 weeks gestation) and *n* = 29 (10.7%) with low birth weight (< 2,500 g) infants. For birth length, *n* = 4 (1.5%) and *n* = 52 (19.2%) were classified as small and large for gestational age, respectively. For head circumference, these were *n* = 14 (5.2%) and *n* = 30 (11.1%). There were no significant differences between MeHg concentrations by preterm birth (vs. not), low birth weight (vs. not), or small for gestational age categories (vs. appropriate for gestational age) in bivariate analyses compared to ln(MeHg) using either *t*-tests, one-way analysis of variance, or unadjusted regression models (data not shown). Exclusion of preterm or low-birth-weight infants from regression models did not substantially change our results (data not shown).

**Table 2:** Mean (95% CI) for birth outcomes stratified by MeHg quartile (*n* = 271).

Outcome	Quartiles of cord blood MeHg (μg/L)				Entire population ND–15.4 μg/L
	ND–0.61 (Q1)	0.62–1.04 (Q2)	1.05–1.84 (Q3)	1.85–15.4 (Q4)	
Gestational age (days)	270.0 (266.3, 273.7)	272.1 (268.9, 275.3)	273.2 (270.1, 276.4)	273.7 (270.7, 276.8)	272.3 (270.6, 273.9)
Birth weight (g)	3,171 (3,033, 3,308)	3,215 (3,061, 3,370)	3,275 (3,141, 3,410)	3,186 (3,038, 3,333)	3,212 (3,140, 3,284)
Birth length (cm)	49.7 (49.0, 50.3)	49.8 (49.1, 50.6)	50.3 (49.7, 50.8)	50.3 (49.7, 51.0)	50.0 (49.7, 50.3)
Head circumference (cm)	33.6 (33.1, 34.1)	33.7 (33.2, 34.1)	33.3 (32.9, 33.7)	33.5 (33.0, 33.9)	33.5 (33.3 33.7)
Ponderal index [(g/cm^3^) × 100]	2.57 (2.50, 2.64)	2.58 (2.50, 2.66)	2.57 (2.50, 2.63)	2.48 (2.41, 2.55)	2.55 (2.51, 2.58)
Abbreviations: ND, not detected; Q, quartile.					

**Figure 1:**
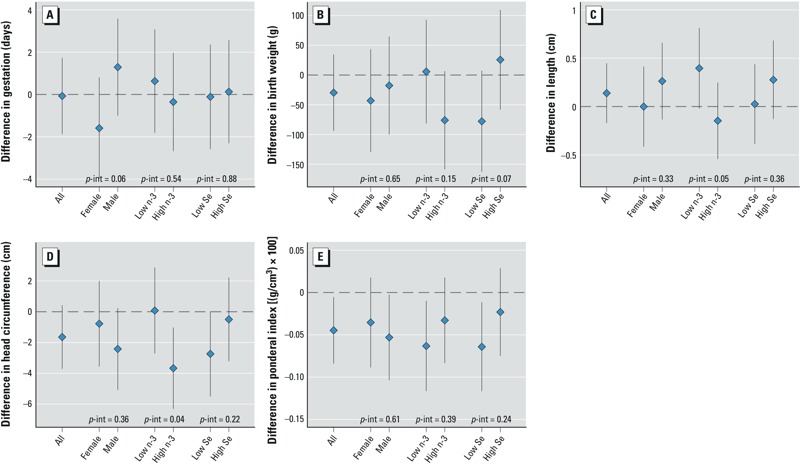
Differences and 95% CIs for birth outcomes related to a 1-unit increase in ln(MeHg), i.e., βln(MeHg). The outcomes include gestational age (days) (*A*); birth weight (g) (*B*); birth length (cm) (*C*); head circumference (cm) (*D*); and ponderal index [(g/cm^3^) × 100] (*E*). Within each panel, from left to right, the lines indicate results from the entire cohort (*n *= 271) and specific subgroups including females (*n *= 121); males (*n *= 150); serum n-3 HUFAS ≤ 50.4 μg/mL, or “low n-3s” (*n *= 136); n-3 HUFAS > 50.4 μg/mL, or “high n-3s” (*n *= 135); serum selenium < 70 μg/L, or “low Se” (*n *= 140); and selenium ≥ 70 μg/L, or “high Se” (*n *= 131). *p*-Values of interaction terms—ln(MeHg) × sex, ln(MeHg) × n-3 HUFAs, and ln(MeHg) × selenium, respectively—are indicated by “*p*-int.”

Multivariable regression model results are presented in Table 3, Figure 1 (see also Supplemental Material, Table S2). In a fully adjusted model for the entire population (model 3), higher MeHg was significantly associated with decreasing ponderal index, but not other outcomes. Adjusting for n-3 HUFAs, selenium, and IHg (i.e., based on model 3 vs. model 2), increased estimated mean differences associated with a 1-unit increase in ln(MeHg) for birth weight (model 2 β = –16.8; 95% CI: –75.0, 41.3; model 3 β = –29.7; 95% CI: –93.9, 34.6) and ponderal index (model 2 β = –0.030; 95% CI: –0.065, 0.005; model 3 β = –0.045; 95% CI: –0.084, –0.005).

**Table 3:** Estimated mean difference in birth outcomes with an increase in cord blood MeHg.

Outcome/model	β for ln(MeHg) (95% CI)
Gestational age (days)
Model 1^*a*^	1.23 (–0.38, 2.84)
Model 2^*b*^	0.86 (–0.81, 2.54)
Model 3^*c*^	–0.07 (–1.87, 1.73)
Birth weight (g)
Model 1^*a*^	–19.58 (–76.14, 36.97)
Model 2^*b*^	–16.84 (–74.96, 41.27)
Model 3^*c*^	–29.65 (–93.89, 34.60)
Birth length (cm)
Model 1^*a*^	0.09 (–0.18, 0.35)
Model 2^*b*^	0.09 (–0.18, 0.37)
Model 3^*c*^	0.14 (–0.17, 0.45)
Head circumference (cm)
Model 1^*a*^	–0.13 (–0.31, 0.06)
Model 2^*b*^	–0.14 (–0.33, 0.04)
Model 3^*c*^	–0.16 (–0.37, 0.04)
Ponderal index [(g/cm^3^) × 100]	
Model 1^*a*^	–0.032 (–0.065, 0.002)*
Model 2^*b*^	–0.029 (–0.065, 0.005)*
Model 3^*c*^	–0.045 (–0.084, –0.005)^#^
^***a***^Model 1 (gestational age adjustments) was adjusted for gestational age (except for gestational age outcome). ^***b***^Model 2 (demographic/medical history adjustments) was additionally adjusted for infant sex (entire cohort only), gestational age (except in gestational age model), maternal age (quadratic), primiparity, prepregnancy BMI, maternal race, maternal smoking, maternal pregestational and gestational hypertension, and maternal pregestational and gestational diabetes. ^***c***^Model 3 (all adjustments) was additionally adjusted for cord serum selenium, cord serum n3-HUFAs, and cord blood IHg. *p < 0.10. ^#^*p* ≤ 0.05. *n *= 271.	

In the Supplemental Material, Table S2 presents data on the effect of additionally including IHg, n-3 HUFAs, or selenium as an additional covariate to model 2. The results suggest that the strength of the relationships between MeHg and birth outcomes was affected by inclusion of n-3 HUFAs or IHg, but not selenium. For example, in gestational age models without n-3 HUFAs or IHg βln(MeHg) was 0.86 days (95% CI: –0.81, 2.54); however, when also adjusting for n-3 HUFAs, βln(MeHg) was 0.38 (95% CI: –1.33, 2.08), or when additionally adjusting for IHg βln(MeHg) was 0.31 (95% CI: –1.54, 2.16). When predicting birth weight using model 2, βln(MeHg) was –16.84 g (95% CI: –74.96, 41.27); however, when IHg was also included as a covariate, βln(MeHg) was –39.67 (95% CI: –103.51, 24.17). Corresponding results in ponderal index models were model 2 βln(MeHg) = –0.030 (g/cm^3^) × 100 (95% CI: –0.065, 0.005), and model 2 + IHg βln(MeHg) = –0.045 (95% CI: –0.084, –0.007).

In stratified analyses (see Supplemental Material, Table S3), we observed a statistically significant association between MeHg with ponderal index among the subgroup of males only [βln(MeHg) = –0.063 (g/cm^3^) × 100, 95% CI: –0.119, –0.007]; between MeHg with head circumference among the subgroup of infants with cord serum n-3 HUFAs > 50.4 μg/mL [βln(MeHg) = –0.30 cm, 95% CI: –0.60, –0.01]; and between MeHg with length among the subgroup of infants with cord serum n-3 HUFAs ≤ 50.4 μg/mL [βln(MeHg) = 0.46 cm, 95% CI: 0.005, 0.91].

Results from interaction models are shown in Figure 1 (see also Supplemental Table S3). Our data suggest there was an interaction between MeHg and n-3 HUFAs with length [βln(MeHg) among those with low n-3 HUFAs = 0.40 cm, 95% CI: –0.02, 0.81; with high n-3 HUFAs = –0.15, 95% CI: –0.54, 0.25; *p*-interaction = 0.048] and head circumference [βln(MeHg) among those with low n-3 HUFAs = 0.01 cm, 95% CI: –0.27, 0.29; with high n-3 HUFAs = –0.37, 95% CI: –0.63, –0.10; *p*-interaction = 0.042]. Additionally, there was a possible interaction between MeHg and sex with gestational age [βln(MeHg) among males = 1.29 days, 95% CI: –1.00, 3.58; with females = –1.59, 95% CI: –3.99, 0.81; *p*-interaction = 0.062] as well as between MeHg and selenium with birth weight [βln(MeHg) among infants with low Se = –77.8 g, 95% CI: –162.9, 7.4; with high Se = 25.8, 95% CI: –58.2, 109.7; *p*-interaction = 0.067].

There were statistically significant associations of selenium with increasing gestational age and increasing birth weight. n-3 HUFAs were significantly associated with decreasing head circumference and infant length. Increasing IHg was related to statistically significant increases in birth weight and ponderal index (data not shown).

The MeHg–ponderal index association in models using categorical variables for either MeHg or IHg was attenuated, but similar to models that used continuous variables for both MeHg and IHg (data not shown). Similarly, an association for total mercury with ponderal index was attenuated, but similar to the association of MeHg with ponderal index (data not shown).

## Discussion

We identified a statistically significant decrease in ponderal index associated with higher cord blood MeHg concentration among newborns in models controlling for selenium, n-3 HUFAs, and IHg. Additionally, our results provide evidence of an interaction of MeHg and n-3 HUFAs with birth length and head circumference.

Our goal was to evaluate the relationship of mercury with prenatal growth accounting for mercury speciation, negative confounding, and potential effect modification by sex. Although we did not observe substantial differences between using total versus MeHg [for ponderal index: βln(totalHg) = –0.044 (g/cm^3^) × 100, 95% CI: –0.099, 0.011; whereas βln(MeHg) = –0.045 95% CI: –0.084, –0.005], or inclusion of confounders, even these small changes could plausibly explain at least part of the mixed results in existing literature.

An Icelandic cohort evaluated fish consumption and ponderal index; however, they did not see a significant association with either fish or fish oil consumption (Thorsdottir et al. 2004). The estimated decrease in ponderal index associated with MeHg in our study population suggests that MeHg exposure during gestation may result in thinner newborns. Although the magnitude of the estimated difference in ponderal index was small, this finding still suggests a potential public health risk among more highly MeHg-exposed infants as well as infants exposed to other risk factors for reduced fetal growth. Barker et al. (2002) first demonstrated how alterations in early development could lead to chronic disease among adults, a concept now understood as the developmental origins of adult disease. Reduced ponderal index is one factor that has been associated with later-life health, particularly cardiometabolic outcomes (Eriksson et al. 2001; Larnkjær et al. 2011).

We did not observe an association of mercury and gestational age, similar to some (Foldspang and Hansen 1990; Grandjean et al. 2001; Lederman et al. 2008), but not all (Ramirez et al. 2000; Xue et al. 2007) reports. Part of this may be explained by population differences or variations in adjustment for confounders. Some studies report that increased fish consumption is related to increased gestational age (Guldner et al. 2007; Heppe et al. 2011; Olsen and Secher 2002). Two studies measuring both cord blood mercury and n-3 HUFAs report a positive association of fatty acids, but no association of mercury with longer gestation (Grandjean et al. 2001; Lucas et al. 2004). Our results additionally suggest that selenium has a small, but significant positive association with gestational age (β = 0.31 days, 95% CI: 0.18, 0.45) and birth weight (β = 6.72 g, 95% CI: 1.71, 11.73).

Much existing literature reports an inverse association (Foldspang and Hansen 1990; Lee et al. 2010; Ramón et al. 2009; Sikorski et al. 1986) or no association (Ding et al. 2013; Grandjean et al. 2001; Lederman et al. 2008; Lucas et al. 2004) of cord blood, maternal blood, or maternal hair mercury with birth weight. Drouillet-Pinard et al. (2010) report increased birth weight with higher maternal hair mercury among infants with overweight mothers; however, this did not persist when they adjusted for fish consumption. It is possible that this result may be explained by collinearity of n-3 HUFAs and mercury in seafood; our data suggest that n-3 HUFAs may confound mercury’s association with birth weight (Table 3; see also Supplemental Material Table S2). This is also supported by studies demonstrating that consumption of different kinds of fish (which likely vary in mercury and n-3 HUFA concentrations) has different associations with birth weight (Guldner et al. 2007; Heppe et al. 2011; Thorsdottir et al. 2004).

Ponderal index and birth weight are correlated in our population (Spearman’s ρ = 0.46); however, babies with higher ponderal index may have lower or average birth weight (if babies have shorter length) and babies with lower ponderal index may have average or higher birth weight (if babies are longer). At this point it would be premature to make clinical conclusions on the basis of these data. Our findings on ponderal index need to be replicated in other studies.

Similar to our study, three cohorts did not report any significant association of cord blood or maternal hair mercury with birth length (Ding et al. 2013; Lederman et al. 2008; Sikorski et al. 1986); there was an increased risk of small for gestational age for length within a Spanish birth cohort with increased cord blood mercury (Ramón et al. 2009); and others found a significant relationship of increasing maternal hair mercury with increased length (Drouillet-Pinard et al. 2010; Gundacker et al. 2010). However, the result from Gundacker and colleagues was unadjusted, and the positive association in findings of Drouillet-Pinard et al. was limited to overweight mothers and did not persist after additionally controlling for seafood consumption (Drouillet-Pinard et al. 2010; Gundacker et al. 2010). Both negative (Halldorsson et al. 2007; Thorsdottir et al. 2004) and positive (Thorsdottir et al. 2004) associations between fish consumption and length at birth have been reported in prior studies.

Several studies reported no association between cord blood or maternal hair mercury with head circumference (Ding et al. 2013; Gundacker et al. 2010; Lederman et al. 2008; Sikorski et al. 1986). Ramirez et al. (2000) report a statistically significant increase in head circumference with cord blood mercury exposure; however, it is unclear what confounders were included in their model. Drouillet-Pinard et al. (2010) report a borderline significant increase in head circumference with increasing maternal hair mercury among overweight mothers; however, this did not persist after adjustment for fish consumption. Contrastingly, decreases in head circumference with increasing hair mercury were observed from the subset of non-overweight mothers among this French cohort and studies that evaluated fish consumption instead of mercury biomarkers (Drouillet-Pinard et al. 2010; Halldorsson et al. 2007; Heppe et al. 2011; Thorsdottir et al. 2004). Although it was not statistically significant, we also observed a decrease in head circumference with increasing MeHg.

We observed a negative association of cord serum n-3 HUFAs with head circumference, length, and birth weight in both our main models as well as our interaction models; this was surprising given the extensive evidence of the beneficial effects of n-3 HUFAs on fetal development (Imhoff-Kunsch et al. 2012). The reason for this is unclear. This result may have been influenced by the relatively low concentrations of n-3 HUFAs found in this population (Lin et al. 2012) or the cross-sectional nature of this analysis (Drouillet et al. 2009). Although n-3 HUFAs and MeHg were not highly correlated (Spearman’s ρ < 0.3), it is still possible that there may have been some influence of collinearity in our analyses. However, our finding of longer gestation is consistent with findings of reduced risk of early preterm delivery with fatty acid supplementation (Imhoff-Kunsch et al. 2012).

In stratified analyses we found MeHg–ponderal index and MeHg–birth weight associations among males, but not females. It is possible that we had insufficient power to detect a relationship among females. However, our results are consistent with previous work. There is evidence that mercury toxicokinetics vary by sex (Ekstrand et al. 2010; Lie et al. 1982; Nielsen 1992). Additionally, there are reports of increased neurologic impacts of mercury exposure among males compared with females (Cauli et al. 2013; Gao et al. 2007; Philibert et al. 2008). We are not aware of previous work looking at sex differences of mercury’s relationship with fetal growth.

Our ability to control for n-3 HUFAs, selenium, and IHg is a strength of this study. The importance of n-3 HUFAs and selenium as negative confounders is that without taking these opposite effects into consideration, one may underestimate mercury’s toxicity (Choi et al. 2008; Karagas et al. 2012; Ou et al. 2014); indeed, our estimates for mercury’s effect on birth weight and ponderal index appear to be strengthened after inclusion of these variables.

In comparison, although it is known that toxicokinetics for MeHg and IHg differ, little is understood regarding this potential impact on fetal growth. Limited prior research with IHg did not identify any association of IHg with birth outcomes (Bashore et al. 2014; Daniels et al. 2007). In contrast, we found that associations of IHg with birth outcomes were generally the inverse of MeHg. These differences may be attributable to the fact that we included both IHg and MeHg as covariates; this raises the possibility that IHg may act as a negative confounder with respect to MeHg exposure. Umbilical cord blood IHg could be the result of placental transport of IHg from the mother to the fetus, or it could be endogenously formed from MeHg demethylation (Carneiro et al. 2014; Ou et al. 2014; Stern and Smith 2003). Therefore, one possible explanation for our results would be that higher concentrations of IHg in umbilical cord serum may be indicative of rapid MeHg metabolism.

Although we had precise speciated mercury measurements, many values were < LOD; therefore, we included observed values < LOD in analyses. Although more precise than a single replacement value, these values are still less precise than higher concentrations; however, the fact that we observed similar results when modeling mercury as quantiles suggests that this was not a major limitation of our study.

Average cord blood mercury concentrations among this study population (either MeHg or total mercury) are lower than concentrations reported in a recent review on low-level exposures (Karagas et al. 2012), except for an Austrian birth cohort (Gundacker et al. 2010). It is possible that in our population these associations were detectable only among the most highly exposed. Another possibility is the association of MeHg with ponderal index or other birth outcomes is nonlinear. Future analyses should consider the nonlinear possibility in their analyses.

This study provides supporting evidence for a relationship between mercury and fetal growth restriction. Our data also suggest that n-3 HUFAs, selenium, and sex may modify these relationships, and that n-3 HUFAs and IHg may confound these relationships. Further consideration of confounding and effect modification should be applied to future work.

## Supplemental Material

(227 KB) PDF
