# Supplementary material for: Cord Blood Methylmercury and Fetal Growth Outcomes in Baltimore Newborns: Potential Confounding and Effect Modification by Omega-3 Fatty Acids, Selenium, and Sex
Source: Environ Health Perspect. 2015 Jun 26;124(3):373–9. doi: 10.1289/ehp.1408596 (PMC4786979; doi:10.1289/ehp.1408596)
Supplement: (227 KB) PDF [file ehp.1408596.s001.acco.pdf]

Environ Health Perspect
DOI: 10.1289/ehp.1408596

Note to Readers: *EHP* strives to ensure that all journal content is accessible to all readers. However, some figures and Supplemental Material published in *EHP* articles may not conform to 508 standards due to the complexity of the information being presented. If you need assistance accessing journal content, please contact ehp508@niehs.nih.gov. Our staff will work with you to assess and meet your accessibility needs within 3 working days.

Supplemental Material

Cord Blood Methylmercury and Fetal Growth Outcomes in Baltimore Newborns: Potential Confounding and Effect Modification by Omega-3 Fatty Acids, Selenium, and Sex

Ellen M. Wells, Julie B. Herbstman, Yu Hong Lin, Jeffery Jarrett, Carl P. Verdon, Cynthia Ward, Kathleen L. Caldwell, Joseph R. Hibbeln, Frank R. Witter, Rolf U. Halden, and Lynn R. Goldman

Table of Contents

Table S1: Spearman correlation coefficients (*p*-values) for mercury, selenium and n-3 HUFAs, THREE Study, 2004-2005
Table S2: Regression coefficient (95% confidence intervals) for MeHg, n-3 HUFAs, selenium, and IHg in various multivariable regression models, N=271, THREE Study, 2004-2005
Table S3: Adjusted regression coefficient (95% confidence intervals) for ln(MeHg), among the full cohort as well as stratum-specific estimates, THREE Study, 2004-2005

Table S1: Spearman correlation coefficients (*p*-values) for mercury, selenium and n-3 HUFAs, THREE Study, 2004-2005

	MeHg	IHg	Total Hg	n-3 HUFAs	Selenium
**MeHg**	1.0				
**IHg**	0.51 (<0.01)	1.0			
**Total Hg**	0.91 (<0.01)	0.53 (<0.01)	1.0		
**n-3 HUFAs**	0.23 (<0.01)	0.15 (0.02)	0.26 (<0.01)	1.0	
**Selenium**	-0.02 (0.70)	-0.07 (0.24)	0.01 (0.85)	0.19 (<0.01)	1.0

THREE = Tracking Health Related to Environmental Exposures; MeHg = methyl mercury; IHg = inorganic mercury; Total Hg = total mercury; n-3 HUFAs = n-3 highly unsaturated fatty acids. N=265 due to some missing values for total mercury.

Table S2: Regression coefficient (95% confidence intervals) for MeHg, n-3 HUFAs, selenium, and IHg in various multivariable regression models, N=271, THREE Study, 2004-2005

Outcome and covariate	Model 2[a]	Model 2[a] + Selenium	Model 2[a] + n-3 HUFAs	Model 2[a] + IHg	Model 3[b]
**Gestational age, days**					
Ln(MeHg), µg/L	0.86 (-0.81, 2.54)	0.87 (-0.73, 2.48)	0.38 (-1.33, 2.08)	0.31 (-1.54, 2.16)	-0.07 (-1.87, 1.73)
Selenium, µg/L		0.32 (0.19 0.46)[#]			0.31 (0.18, 0.45)[#]
n-3 HUFAs, µg/mL			0.13 (0.02, 0.24)[#]		0.07 (-0.03, 0.18)
Ln(IHg), µg/L				0.63 (-0.26, 1.53)	0.77 (-0.09, 1.63)[*]
**Birthweight, grams**					
Ln(MeHg), µg/L	-16.84 (-74.96, 41.27)	-15.51 (-73.25, 42.24)	-7.72 (-67.27, 51.84)	-39.67 (-103.51, 24.17)	-29.65 (-93.89, 34.60)
Selenium, µg/L		5.29 (0.35, 10.23)[#]			6.72 (1.71, 11.73)
n-3 HUFAs, µg/mL			-2.57 (-6.34, 1.20)		-3.61 (-7.40, 0.17)[*]
Ln(IHg), µg/L				26.40 (-4.66, 57.46)[*]	31.62 (0.76, 62.48)[#]
**Length, cm**					
Ln(MeHg), µg/L	0.09 (-0.18, 0.37)	0.10 (-0.18, 0.37)	0.15 (-0.13, 0.43)	0.08 (-0.22, 0.39)	0.14 (-0.17, 0.45)
Selenium, µg/L		0.01 (-0.01, 0.04)			0.02 (-0.01, 0.04)
n-3 HUFAs, µg/mL			-0.02 (-0.03, 0.002)[*]		-0.02 (-0.04, -0.0001)[#]
Ln(IHg), µg/L				0.01 (-0.14, 0.16)	0.03 (-0.12, 0.18)
**Head circumference, cm**					
Ln(MeHg), µg/L	-0.14 (-0.33, 0.04)	-0.14 (-0.33, 0.04)	-0.10 (-0.29, 0.09)	-0.21 (-0.42, -0.01)[#]	-0.16 (-0.37, 0.04)
Selenium, µg/L		0.005 (-0.01, 0.02)			0.01 (-0.01, 0.03)
n-3 HUFAs, µg/mL			-0.01 (-0.03, -0.002)[#]		-0.02 (-0.03, -0.003)[#]
Ln(IHg), µg/L				0.08 (-0.02, 0.18)	0.09 (-0.01, 0.19)[*]
**Ponderal index, $(g/cm^3)\times100$**					
Ln(MeHg), µg/L	-0.030 (-0.065, 0.005)[*]	-0.029 (-0.064, 0.006)	-0.030 (-0.066, 0.007)	-0.045 (-0.084, -0.007)[#]	-0.045 (-0.084, -0.005)[#]
Selenium, µg/L		0.002 (-0.001, 0.005)			0.003 (-0.001, 0.006)
n-3 HUFAs, µg/mL			-0.00003 (-0.002, 0.002)		-0.0004 (-0.003, 0.002)
Ln(IHg), µg/L				0.018 (-0.001, 0.037)[*]	0.020 (0.001, 0.039)[#]

THREE = Tracking Health Related to Environmental Exposures; HUFA = highly unsaturated fatty acid; MeHg = methylmercury; IHg = inorganic mercury; HUFA = highly unsaturated fatty acid; CI = confidence interval.

Model 2 includes the following covariates: methylmercury, gestational age (except for gestational age outcome), infant sex (entire cohort only), maternal age (quadratic), primiparity, prepregnancy body mass index, maternal race, maternal smoking, maternal pregestational and gestational hypertension, and maternal pregestational and gestational diabetes.

Model 3 includes the covariates included in model 2 as well as selenium, n-3 HUFAs, and IHg.

[*]*p*-value <0.10; [#]*p*-value ≤0.05.

Table S3: Adjusted[a] regression coefficient (95% confidence intervals) for ln(MeHg), among the full cohort as well as stratum-specific estimates, THREE Study, 2004-2005

Cohort	β (95% CI) or *p*-value	Gestational age, days	Birthweight, grams	Length, cm	Head circumference, cm	Ponderal index, (g/cm³)×100
Full cohort	β for ln(MeHg)	-0.07 (-1.87, 1.73)	-29.6 (-93.9, 34.6)	0.14 (-0.17, 0.45)	-0.16 (-0.37, 0.04)	-0.045 (-0.084, -0.005)#
Males	β for ln(MeHg)	1.42 (-1.21, 4.05)	-13.5 (-110.2, 83.3)	0.35 (-0.06, 0.75)*	-0.29 (-0.61, 0.020)*	-0.063 (-0.12, -0.007)#
Females	β for ln(MeHg)	-1.68 (-4.18, 0.83)	-38.5 (-124.2, 47.3)	-0.02 (-0.52, 0.47)	-0.04 (-0.30, 0.22)	-0.029 (-0.086, 0.027)
Full cohort, interaction with sex	β for ln(MeHg)	-1.59 (-3.99, 0.81)	-43.1 (-129.6, 43.5)	-0.0004 (-0.42, 0.41)	-0.08 (-0.36, 0.20)	-0.035 (-0.088, 0.018)
	β for sex	0.48 (-2.66, 3.63)	148.8 (35.8, 261.8)#	0.60 (0.06, 1.14)#	0.32 (-0.04, 0.69)*	0.018 (-0.051, 0.087)
	β for sex*ln(MeHg)	2.88 (-0.15, 5.91)*	25.5 (-84.2, 135.2)	0.26 (-0.26, 0.79)	-0.16 (-0.52, 0.19)	-0.018 (-0.085, 0.050)
	p-value (interaction)	0.062	0.648	0.325	0.360	0.605
Low n-3 HUFAs[b]	β for ln(MeHg)	0.21 (-2.54, 2.95)	25.2 (-71.0, 121.4)	0.46 (0.005, 0.91)#	0.001 (-0.32, 0.32)	-0.056 (-0.113, 0.001)*
High n-3 HUFAs[b]	β for ln(MeHg)	-1.32 (-3.76, 1.12)	-88.4 (-185.8, 8.9)*	-0.20 (-0.68, 0.28)	-0.30 (-0.60, -0.01)#	-0.038 (-0.097, 0.022)
Full cohort, interaction with n-3 HUFAs[b]	β for ln(MeHg)	0.63 (-1.81, 3.08)	5.8 (-81.5, 93.0)	0.40 (-0.02, 0.81)*	0.01 (-0.27, 0.29)	-0.063 (-0.117, -0.010)#
	β for n-3 HUFAs	0.96 (-2.24, 4.16)	-68.4 (-182.5, 45.7)	-0.51 (-1.06, 0.03)#	-0.41 (-0.77, -0.04)#	0.017 (-0.053, 0.086)
	β for n-3 HUFAs*ln(MeHg)	-0.99 (-4.14, 2.17)	-81.7 (-194.4, 30.9)	-0.54 (-1.08, -0.01)*	-0.37 (-0.74, -0.01)#	0.030 (-0.039, 0.099)
	p-value (interaction)	0.538	0.154	0.048	0.042	0.386
Low Se[c]	β for ln(MeHg)	-0.35 (-3.55, 2.85)	-47.2 (-133.9, 39.6)	0.08 (-0.37, 0.54)	-0.25 (-0.60, 0.09)	-0.050 (-0.105, 0.005)*
High Se[c]	β for ln(MeHg)	-0.07 (-2.00, 1.86)	-8.0 (-106.2, 90.1)	0.25 (-0.19, 0.68)	-0.07 (-0.32, 0.19)	-0.040 (-0.100, 0.021)
Full cohort, interaction with Se[c]	β for ln(MeHg)	-0.11 (-2.58, 2.36)	-77.8 (-162.9, 7.4)*	0.03 (-0.39, 0.44)	-0.27 (-0.55, 0.003)*	-0.064 (-0.117, -0.012)#
	β for Se	4.36 (1.01, 7.71)#	182.0 (64.9, 299.0)#	0.58 (0.02, 1.15)#	0.20 (-0.17, 0.58)	0.061 (-0.011, 0.134)*
	β for Se*ln(MeHg)	0.24 (-2.98, 3.46)	103.5 (-7.3, 214.4)*	0.25 (-0.28, 0.77)	0.22 (-0.14, 0.58)	0.041 (-0.027, 0.109)
	p-value (interaction)	0.883	0.067	0.357	0.221	0.238

MeHg = methylmercury; IHg = inorganic mercury; HUFAs = highly unsaturated fatty acids; THREE = Tracking Health Related to Environmental Exposures; CI = confidence interval; Se = selenium.

Full cohort n=271; females only n=121; males only n=150; low Se only n=140; high Se only n=131; low n-3 HUFAs only n=136; high n-3 HUFAs only n=135.

[a]Models adjusted for gestational age (except for gestational age outcome), infant sex (except for models stratified by sex), maternal age (quadratic), primiparity, prepregnancy body mass index, maternal race, maternal smoking, maternal pregestational and gestational hypertension, maternal pregestational and gestational diabetes, cord serum selenium, cord serum n-3 HUFAs, and cord blood IHg..

[b]Low cord serum n-3 HUFAs: ≤ 50.4 μg/mL; high cord serum n-3 HUFAs: > 50.4 μg/mL.

[c]Low cord serum selenium: < 70 μg/L; high cord serum selenium: ≥ 70 μg/L.

*$p < 0.10$; # $p < 0.05$.
